# Commentary on “Drivers of health in sub-Saharan Africa”. A dynamic panel analysis

**DOI:** 10.1016/j.hpopen.2021.100034

**Published:** 2021-01-21

**Authors:** Mwoya Byaro

**Affiliations:** IRDP, Research and Publication Unit, P.O BOX 11957, Mwanza, Tanzania

## Abstract

• This commentary discuss the drawbacks observed in the published article in Health Policy OPEN.
• It explains the common problems overlooked by the scholars and reviewers when applied dynamic panel data model.
• Too many instruments counts in GMM estimators leads to the problem of instrument proliferation.
• The solution to reduce the number of instruments in GMM estimators are addressed.

I have read with interest the published article in *Healthy Policy Open (1:2020; 100013)* by two outstanding scholars [1], “Drivers of health in sub-Saharan Africa” a dynamic panel analysis. The authors identified the key drivers of health in sub-Saharan Africa to include increases in health expenditure, education attainment, health care access quality, urbanization, per capita income growth and access to clean water. Likewise, the findings suggest reductions in HIV prevalence and excessive alcohol consumptions in the region. Their findings were robust due to different diagnostic tests applied such as first and second-order serial correlation (AR) and Sargan test of over-identifying restriction [1]. Overall, their findings were also consistent with other empirical literature.

The authors used dynamic panel data model which is popular and mainly characterized by two features. The first feature includes the lagged dependent variable on the right hand side, availability of lags of the endogenous variables and another feature is characterized by having a panel data with both cross sectional and time series dimension [2], [3].

In this commentary, I write to inform my colleagues about the number of instruments used in their article (i.e. Difference Dynamic Panel model, DPD) based on the Generalized Methods of Moments (GMM) justified to control endogeneity of variables in the regression model. It’s true that the first difference GMM was applied to eliminate the fixed effects as shown in Eqs. (2) and (3). However, the GMM estimators are inconsistent as the number of instruments becomes too large as outlined by authors in Table 1 (i.e. 99 and 87). This is proved by the *p-value* shown by authors indicating Sargan test with probability value (i.e. *p-value* = 1.000). One of the possible reasons for the authors to have large *p-value* in Sargan test is that, the number of instruments used are too many or large (i.e. 99 instruments for life expectancy and 87 instruments for infant mortality) as revealed in the article. Roodman [4] asserts that too many instruments in the dynamic panel data GMM can over fit the endogenous variable and leading to biased coefficient estimates. In turn, Bontempi and Mammi, [3] alerts scholars on the drawbacks of implementing DPD models by GMM with many instruments. To address this challenge, Roodman [4] recommended reducing the number of instruments by setting *collapse* option using *xtabond2*. Similarly, Bontempi and Mammi [3] introduced another statistical strategy to reduce the instrument count in panel GMM. This strategy requires the implementation of the new command *pca2* based on the principal component analysis known as “principal components instrumental variables reduction” [3]. In this views, the authors Mwimba Chewe and Peter Hangoma [1], were supposed to reduce the instrument count from either 99 instruments for life expectancy or 87 instruments for infant mortality to at least lower than 30 number of groups/countries [see [3]]. This may help to reduce the bias in parameter estimates and its associated *p-value* in the Sargan test. In general, the outcome of the test for over-identifying restrictions (Sargan test) reported by authors [1], was weakened by the high instruments count [see [3]].

The take home message to the readers, scholars and reviewers is that, the problem of instrument proliferation in DPD models using GMM estimators are often overlooked in many empirical analysis (See for example, [1], [5], [6]). Therefore, this commentary provides the requirements for scholars and other reviewers for future studies to highlight the importance of instruments count check in case the models employ the dynamic GMM estimators.

## CRediT authorship contribution statement

**Mwoya Byaro:** Writing - review & editing.

## Declaration of Competing Interest

The authors declare that they have no known competing financial interests or personal relationships that could have appeared to influence the work reported in this paper.
